# Dental Caries among Patients Visiting the Dental Outpatient Department in a Tertiary Care Centre: A Descriptive Cross-sectional Study

**DOI:** 10.31729/jnma.8215

**Published:** 2023-07-30

**Authors:** Bhawana Karki, Simran Kunwar, Gitu Gaire, Kalpana Roka Magar, Laxmi Bhusal, Prajani Giri, Krishna Subedi

**Affiliations:** 1 Department of Community Dentistry, Gandaki Medical College Teaching Hospital and Research Centre, Pokhara, Kaski, Nepal

**Keywords:** *dental caries*, *oral hygiene*, *prevalence*

## Abstract

**Introduction:**

Dental caries is an alarming public health problem globally causing significant morbidity and mortality. Early diagnosis and prompt treatment help in the prevention of complications. The aim of this study was to find out the prevalence of dental caries among patients visiting the dental Outpatient Department in a tertiary care centre.

**Method:**

A descriptive cross-sectional study was conducted among patients visiting the dental Outpatient Department in a tertiary care centre. Data collection was done from 15 November 2022 to 15 February 2023 after taking ethical approval from the Institutional Review Committee (Reference number: 236/078/079). All patients visiting the dental Outpatient Department above 18 years of age who provided written informed consent were included in this study. Convenience sampling was done. Point estimate and 95% Confidence Interval were calculated.

**Results:**

Among 270 patients, the prevalence of dental caries was 214 (79.26%) (74.42-84.10, 95% Confidence Interval).

**Conclusions:**

The prevalence of dental caries among patients was higher than other studies done in similar settings.

## INTRODUCTION

Dental caries is one of the most prevalent chronic diseases in human beings that varies with age, sex, dietary habits, socioeconomic status, and poor oral hygiene status.^1^ If left untreated, it can lead to pain, infection, tooth loss, and other long-term effects.^2^

Worldwide, it is estimated that around two billion people suffer from caries of permanent teeth.^3^ In Nepal, the prevalence of caries in adults of age 35-49 years old is 57.5%.^4^ Dental caries is an alarming public health problem globally causing significant morbidity and mortality.^5^ However, only a few studies have been published.

Therefore, this study aimed to find out the prevalence of dental caries among patients visiting the dental Outpatient Department (OPD) in a tertiary care centre.

## METHODS

This descriptive cross-sectional study was conducted among patients presenting to the dental OPD of Gandaki Medical College Teaching Hospital and Research Centre, Pokhara, Kaski, Nepal after obtaining ethical approval from the Institutional Review Committee (Reference number: 236/078/079). Data collection was done from 15 November 2022 to 15 February 2023. All patients visiting dental OPD above 18 years of age who provided written informed consent were included in this study. Mentally disabled patients were excluded as it is difficult for them to provide correct information. Convenience sampling was done. The sample size was calculated using the formula:


$$\begin{array}{ll} \text{n} & ={\text{Z}}^{2}\times \frac{\text{p}\times \text{q}}{{\text{e}}^{\text{2}}}\\ & =1.96^{2}\times \frac{0.64\times 0.36}{0.06^{2}}\\ & =246 \end{array}$$

Where,

n = minimum required sample size
Z = 1.96 at 95% Confidence Interval (CI)
p = prevalence of dental caries taken from a similar study done in past, 64.41%^6^
q = 1-p
e = margin of error, 6%

The calculated sample size was 246. After adding a 10% non-response rate, the sample size was 270.

A self-structured questionnaire was prepared in English and Nepali languages which included the patients' oral hygiene methods, dietary habits, and dental visit history. Investigators took face-to-face interviews with the patients recording the patient's general information which included the patient's name, age, sex, occupation, education level and income.

Clinical examination was performed under artificial light using plane mouth mirrors and explorers. Instruments used during data collection were sterilized before the dental examination and at the end of the data collection period in the dental hospital. Disposable gloves and masks were used during the data collection. Data collection was done by using the decayed, missing, and filled teeth (DMFT) index given by the World Health Organization modification of dental caries criteria (1987).^7^

Data were collected and analyzed using IBM SPSS Statistics version 21.0. Point estimate and 95% CI were calculated.

## RESULTS

Among 270 patients, the prevalence of dental caries during the study period was 214 (79.26%) (74.53-84.19, 95% CI). The total mean DMFT score was 5.44±4.75 (Table 1).

**Table 1:** Mean DMFT (n=214).

Parameters		Mean±SD
Sex	Male	5.01±4.66
	Female	5.77±4.81

**Figure 1:**
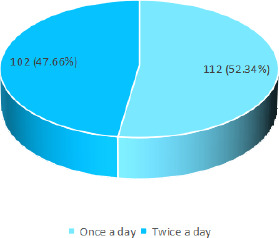
Dental caries according to tooth brushing habits (n= 214).

Among 214 patients, dental caries was seen in 112 (52.34%) patients who brushed their teeth once daily (Figure 1).

Among 214 patients with dental caries, 128 (47.41%) females were affected. The mean age was found to be 43.12±16.26 years (Table 2).

**Table 2:** Demographic details (n = 214).

Parameters		n (%)
**Sex**	Male	86 (31.85)
	Female	128 (47.41)
	Illiterate	30 (14.01)
**Educational status**	Primary level	18 (8.41)
	Secondary level	72 (33.64)
	Higher secondary level	43 (20.09)
	Bachelor and above	51 (23.83)
	Farmer	33 (15.42)
	Housewife	65 (30.37)
	Student	39 (18.22)
**Occupation**	Retired	25 (11.68)
	Self-employed	23 (10.74)
	Job holder	29 (13.55)

Among 214 patients with dental caries, 212 (99.06%) used toothbrush and toothpaste 210 (98.13%) to clean their teeth. The majority of the patients 116 (54.20%) did not know whether their toothpaste was fluoridated or not and did not use any inter-dental aids 170 (79.43%) to clean their teeth (Table 3).

**Table 3:** Different parameters of oral hygiene (n= 214).

Tools for cleaning teeth	n (%)
Toothbrush	212 (99.06)
Finger	2 (0.93)
**Time of brushing teeth**	
Before food	82 (38.31)
After food	91 (42.52)
Before food in the morning and after food in the evening	41 (19.15)
**Materials for brushing teeth**	
Toothpaste	210 (98.13)
Tooth powder	3 (1.40)
Others	1 (0.046)
**Use of fluoridated toothpaste**	
Yes	81 (37.85)
No	17 (7.94)
Do not know	116 (54.20)
**Use of inter-dental aids**	
Yes	44 (20.56)
No	170 (79.43)
**The habit of rinsing after every snacking**	
Yes	116 (54.20)
No	98 (45.79)
**Frequency of consumption of sugary foods**	
Once daily	41 (19.15)
Twice daily	68 (31.77)
Thrice daily	37 (17.28)
Thrice daily or more	23 (10.74)
Once or twice in a week	22 (10.28)
Zero	23 (10.74)
**Last dental visit history**	
Six months ago	68 (31.77)
One year ago	92 (42.99)
First time	43 (20.09)
I do not remember	11 (5.14)
**Reason for visiting the dentist**	
Pain	69 (32.24)
Swelling of the gums	12 (5.60)
Dental Caries	70 (32.71)
Consultation	24 (11.21)
Others	8 (3.73)

## DISCUSSION

The prevalence of dental caries among patients visiting the dental OPD was found to be 214 (79.26%) which is higher than in similar studies. In a study done in a tertiary care centre in Pokhara, Nepal, the prevalence was found to be 64.41%.^6^ Our results were similar to a cross-sectional study done among patients attending general hospital in Ethiopia where the prevalence of caries was found to be 78.2%.^8^ The similarity with this study could be because both are hospital-based studies where there might be a high prevalence of disease as compared to the community level. The higher prevalence in our study compared to other studies could have been because the mean age in our study was found to be higher than other studies (42.74±16.42) and as evidence shows; with increased age, exposure to caries is increased.^9^

The mean DMFT score in our study was 5.44±4.75 which is significantly higher than studies done in Nepal, China and Africa.^9-11^ This difference might be due to the study population variation and the socio-demographic difference between the countries.

The prevalence of dental caries was high in female patients as compared to males. Similar results were seen in studies in Nepal, China and Africa.^6,9,11^ Higher prevalence in females can be attributed to hormonal fluctuations during events such as menstruation, puberty and pregnancy making the oral environment more prone to caries.^11,12^ Earlier eruption of teeth in girls, easier access to food supplies by women, and frequent snacking during food preparation and pregnancy aid in increased prevalence of dental caries in women.^13^

Dental caries was found to be more prevalent among those who brushed their teeth once daily than those who brushed their teeth twice daily. This was also similar to a study done in East Africa where participants with poor oral hygiene practice were 1.96 times more prone to develop dental caries.^11^ This can be attributed to inadequate removal of the microbial bio-films, and food debris at one brushing which makes them more prone to dental caries.^11^

Among 214 patients with dental caries, almost all of them used toothbrushes 212 (99.06%) and toothpaste 210 (98.13%) to clean their teeth which is higher than a study done in Nepal where 86.9% used toothbrushes and toothpaste.^14^ The significant number of patients 170 (79.43%) did not use any inter-dental aids to clean their teeth which were higher than a study done in Iran (53.7%).^15^ Majority of the patients 133 (62.14%) neither used nor knew about fluoridated toothpaste which is higher than the study done in Nepal (27.4%).^14^ The discrepancy may be attributed to the availability and affordability of dental floss and inter-dental brush along with poor awareness about these items.

As the data was taken from a single centre, thus the results of the study cannot be generalized to the whole population. It does not represent the burden of the dental caries in the country. Although, it does give an insight into the caries prevalence, oral health knowledge and preventive practice are needed in this region.

## CONCLUSIONS

The prevalence of dental caries was found to be higher than in other studies done in similar settings. Therefore, local policies should be emphasized on the high prevalence of dental caries. Oral health policies and programs should be oriented toward addressing dental caries which will uplift the quality of oral health of people.
